# The Hospital Nursing Supplement

**Published:** 1896-10-10

**Authors:** 


### "The Hospital" Nursing Mirror.

Being the Extra Nursing Supplement of "The Hospital" Newspaper.

[Contributions for this Supplement should be addressed to the Editor, The Hospital, 28 & 29, Southampton Street, Strand, London, W.C., and should have the word "Nursing" plainly written in left-hand top corner of the envelope.]

### News from the Nursing World.

### PRINCESS CHRISTIAN AND NURSES' UNIFORMS.

No one of our Royal Princesses has interested herself more than Princess Christian in matters connected with nursing, and it is rather too bad that anything so silly as the statement that Her Royal Highness "is in favour of abolishing the uniforms of nurses" should be just now making food for "copy" in the newspapers. The argument for this "abolition" is said to be based upon the conviction that "the dress is one of the chief inducements to women to go through the preliminary probation," a remark very unlikely to have emanated from one so practically acquainted with nurses themselves as the Princess. So long as discipline and order reign in hospital wards, so long will nurses wear a uniform dress; and the prettier and more becoming it is, while retaining the utmost simplicity and neatness, the better. The question of outdoor uniform is very different, but its convenience has at all times much to recommend it, and we fancy this reason is more likely to keep it popular amongst nurses than any notion of vanity.

### LADY DUFFERIN'S FUND.

The sixty-first meeting of the Bengal branch of Lady Dufferin's Fund was lately held at Belvidere, the chair being taken by the Lieutenant-Governor. A letter from the Hon. Nawab Syed Hossein Khan Bahadur, C.I.E., announced his intention of giving a donation to the Bengal branch of the fund of 25,000 rupees.

### FLORENCE NIGHTINGALE "TO THE NURSES."

The thirty-sixth quarterly letter to the Mary Adelaide Nurses, by Mrs. Roundell, is a most interesting little volume, not only because it is a charmingly written biography of that noble woman, Agnes Jones, the story of whose life and early death in the midst of the work she loved, ought to be studied by every nurse, but because it also contains a supplement by Miss Nightingale, and two extracts therefrom in facsimile as it was written by her own hand only this August, "To the Nurses." The Archbishop of Armagh, who, as rector of Fahan, the home of Agnes Jones' girlhood, knew her well, contributes a few recollections of her in those early days. A daintily got up reprint of the little book, which all nurses should read, is published, at the price of sixpence, by Messrs. Bickers and Son, Leicester Square.

### A NEW IDEA.

The Newry Board of Guardians have hit upon an ingenious plan whereby to save their pockets. At a recent meeting the following resolution was passed: "That we look upon the appointment of trained nurses for the workhouse hospitals as an increase to the medical staff, for the part payment of which the Government give an annual grant, and that, as these appointments were made in most instances upon the imperative orders issued by the Local Government Board, we consider that it is only common justice to the ratepayers that the salaries of these officials be paid from an Imperial source. That copies of the resolution be forwarded to the Lord Lieutenant and Chief Secretary, and that they be requested to use their influence with the Lords of the Treasury to have the salaries paid to the trained nurses in workhouse hospitals in Ireland defrayed out of the Government grant, at present confined to the payment of medical and educational expenses incurred in the management of Irish workhouses."

### A COMPETITION FOR CHILDREN.

A painting competition has been arranged in aid of the Children's Hospital, Temple Street, Dublin. By applying to the matron one of the outline painting books delineating scenes of childhood will be sent for the price of one shilling. Prizes from ten shillings to three guineas are offered for the best production from the brush of children and their elders, according to the class of competition entered for, and all books must be completed by December 8th. The Moy Bell painting book is capable of making a charming little picture book in skilful hands. The hospital is in debt, and it is intended to hold a *fête* in its behalf in the spring of next year. In connection with this, doll dressing and pinafore making competitions are to be held.

### ROYAL ALEXANDRA HOSPITAL, BRIGHTON.

A two days' bazaar was opened by Lady Gage at the Royal Alexandra Hospital for Sick Children, Brighton, on September 25th, whilst on the second day Lady Thomas presided. The proceeds are to be devoted to the fund for the completion of the nurses' "Dormitory" just erected in the grounds, and we are glad to see they amounted for the two days to a total of £202 18s. 8d.

### A WOMEN'S HOSPITAL FOR EDINBURGH.

Miss Sophia Jex-Blake, M.D., Dean of the Edinburgh School of Medicine for Women, is appealing to the public for help in raising £10,000 or £12,000 "with which to erect and furnish a thoroughly good women's hospital in Edinburgh," to be the first of its kind in Scotland, worked entirely by medical women.

### A SUCCESSFUL BAZAAR.

The bazaar held recently at Winchcombe Cottage Hospital was a thorough success, thanks to the energy of the stall holders and Mrs. McCaul, the matron, who acted as honorary secretary for the occasion. The small sum of £10 will cover expenses, and deducting this, the hospital will benefit to the very creditable sum of £100.

### FLOWERS FOR THE HOSPITALS.

Now is the time that bedding-out plants from the Royal Parks are to be had for the asking, and application should be made at Hampton Court, and Regent's and Hyde Parks for plants which, put into pots, might serve to brighten hospital wards through the coming winter. The geraniums and marguerites which have made London window-boxes gay during the summer months would also be thankfully received at many a hospital or workhouse infirmary, where a little greenery on the ward table or in the windows is a real pleasure to the inmates of these institutions.

### A REMINDER.

Communications of current interest from our readers are always welcome, and we are glad to publish notice of appointments "in this week's Hospital" whenever possible; but we should be able to please our contributors by a more prompt insertion if they would remember to send letters containing such news to the office (28 & 29, Southampton Street, Strand, London, W.C.), as early in the week as possible. We fancy our readers feel somewhat injured if they do not get their Hospital on Friday morning. It stands to reason, therefore, that letters which do not reach us till Wednesday stand a poor chance of receiving attention that same week.

### NURSING AT RIO.

The recent troubles between the authorities and the nursing staff at the Strangers' Hospital, Rio de Janeiro, to which we drew attention in these columns on August 8th last, and which resulted in the resignation of several of the nurses, it will be remembered were stated to have arisen because the nurses objected to being sent out to private cases, an arrangement not defined in their agreement. The committee of the hospital have now decided in future engagements with nurses to expressly specify "out nursing" as one of the terms of service, so that those who enter on such service will do so with their eyes open on this matter. We believe the matron of the Strangers Hospital is now in England engaging a fresh staff. It will be well, then, that nurses should come to some understanding as to this matter before entering on any engagement, and, in fact, in regard to any foreign appointment it is always well to have the exact terms of service put in writing and properly signed.

### CONVERSAZIONE AT ST. MARY'S HOSPITAL.

The conversazione held at the St. Mary's Hospital in aid of the building fund last Friday evening was in every way a great success, and most enjoyable. The refreshment and supper rooms were largely patronised, and all the ladies in attendance were unflagging in their endeavours to make the evening enjoyable. A large number of guests were present. During the evening the band of the Grenadier Guards played at intervals; the Queen's harpist, Mr. John Thomas, played; there were recitations and songs by well-known artistes; living pictures, and a demonstration of the Röntgen Rays; magic-lantern views formed the principle attractions of the evening.

### VISITORS FROM FRANCE.

Under the guidance of Miss Williams, teacher of English in the École Normale, Paris, a number of ladies interested in the subject of elementary education have been visiting London, sent over to England by the French Government. During their stay they went to see the London School of Medicine for Women, accompanied by M. le Dr. Physsalix and Madame Physsalix, who is herself studying medicine in Paris, and visited the Royal Free Hospital and the New Hospital for Women. Mrs. Marshall, M.D., Mrs. Garrett Anderson, M.D., and Mrs. Thorne received them at the New Hospital, and showed the party also over the school in Handel Street. At the Royal Free Hospital Mr. Thies and Miss Wedgwood conducted them round the wards, and over the nurses' and students' quarters. The French ladies were very much interested in all they saw.

### A RECOGNITION OF GOOD SERVICE.

At a recent meeting of the Newton Board of Guardians, Colonel Walcott, C.B., proposed, "in the cause of economy," that the salary of the head nurse be increased by £5; and a lady Guardian, in seconding, spoke with appreciation of the nurse's "skilful attendance and cheerful kindness," adding that her work had been done "without the slightest friction." The Guardians are wise in showing recognition of good service, and we are glad to see that, in spite of some opposition, ultimately the increase was granted on a division of the Board by twenty-seven votes to eighteen.

### EXTENSION OF NURSES' HOME, BRISTOL GENERAL HOSPITAL.

The opening of the new wing to the Nurses' Home of the Bristol General Hos pital on September 28th was celebrated by an At Home at the hospital. Mr. Joseph Storrs Fry, the treasurer, made a short speech, in which he said he felt sure the subscribers would agree that, in providing these conveniences for their nursing staff, the governors were only doing their duty, because all who supported the hospital would wish that everything reasonable should be done for the comfort of this most excellent branch of their workers. The success of the institution depended in a large measure upon the character of the work done by the nurses, so it was essential that they should be properly housed and cared for. He took that opportunity of thanking Miss Morris, their excellent matron, and her assistants for the admirable way in which they supported the medical staff. The new building provides twenty additional bed-rooms and an extra sitting-room, with bath-rooms on each floor, and a large box-room in the basement.

### SOCIETY OF TRAINED MASSEUSES.

The next examination of this society will be held at the Trained Nurses' Club, 12, Buckingham Street, Strand, W.C., on Wednesday and Thursday, October 14th and 15th. Candidates wishing to present themselves should send in their names at once without loss of time. We have received many inquiries lately as to "where massage is taught in London," and "what examinations are there to pass?" and may call attention to the fact that Mrs. A. Arthur, hon. secretary of the Society of Trained Masseuses, will give all particulars. Letters, endorsed "Massage, to be forwarded," to be sent to her at the office, 12, Buckingham Street. A stamped envelope should be enclosed for reply. Personal interviews can be had by appointment on Friday mornings.

### SHORT ITEMS.

Miss Alström, Miss Joyce, Miss Shepheard, and Miss Stewart Lane have passed the first M.B. examination at the Durham College of Medicine, Newcastle-on-Tyne. Miss Stewart has also passed her second M.B.—A scheme is on foot to place a portrait of the late Dr. A. F. Wood in the nurses' dining-room at the Edinburgh City Hospital.—The collections at the Honiton annual carnival, which is to take place on October 29th, will be in aid of the Devon and Exeter Hospital and the Honiton District Nursing Association.—The date of the bazaar announced last week to take place on November 26th at St. Andrew's Hospital, Clewer, has been postponed to St. Andrew's Day, November 30th.

### Hygiene: For Nurses.

By John Glaister, M.D., F.F.P.S.G., D.P.H.Camb., Professor of Forensic Medicine and Public Health, St. Mungo's College, Glasgow, &c.

### XXVII. — SEWAGE DISPOSAL — PRECIPITATION — IRRIGATION — FILTRATION — HOUSE REFUSE DISPOSAL — CONSERVANCY SYSTEMS — PRACTICAL HINTS FOR HOUSEHOLDERS AND NURSES.

Disposal of Sewage.—The proper disposal of the water-borne sewage of a populous place ought to be a matter of solicitude on the part of sanitary authorities. Hitherto but little attention has been paid, in many cases, to the fitness of place and mode of disposal. Not infrequently, if the place be situated on a river, a canal, or by the seashore, the main outlet sewers are simply turned into the waterway to pollute the river, canal, or foreshore, which is a contravention of the Rivers Pollution Act. The Clyde at Glasgow, the Liffey at Dublin, the Seine below Paris, and other rivers, have become converted into elongated sewers, seething with fermenting corruption. It has often been alleged that such practice and results are not positively injurious to health. This is, however, contrary both to common sense and fact; the only difficulty is to bring the proof home. That sewage can be satisfactorily disposed of in relation to a river has been demonstrated in the case of London and elsewhere. The chief objection to the initiation of like schemes in other cities is the financial one, but what citizens spend in this way they are likely to save by healthier and longer lives. All applied methods for sewage disposal may be divided into three kinds, viz.: (1) by chemical treatment or precipitation, (2) by irrigation, and (3) by filtration. The first mode consists in mixing with the sewage by mechanical means certain chemical substances in certain definite proportions, allowing the mixture to settle in settling-ponds, running off the clear supernatant fluid, sometimes passing this through filter-beds, and ultimately discharging the clear effluent into a river or other water-way. Lime, alumina, and iron are the principal precipitants now employed, and they act both chemically and mechanically. The solid portion or sludge is, if possible, sold as manure, either in bulk or compressed in the form of cakes. The second mode is adopted where, in the neighbourhood of a town, suitable land is easily and cheaply procurable. The sewage is conveyed to this land, and is run upon the surface of the sloping ground in channels which distribute it equally. Porous sandy soil is the best, since percolation quickly occurs. The solids are incorporated into the soil by agricultural operations. The percolated water finds its way into natural channels, just as the rain does. The land, so enriched, is utilised for the cultivation of certain kinds of crops.

The third mode is by filtration. To a considerable extent this happens also in the second mode. But in this process, where filtration is the principal feature in the clarification of the sewage, suitable land must be prepared after the fashion of a large filter-bed, which is divided into a series of plots. The bottom of the bed—six feet from the surface—is laid at intervals with agricultural drain-pipes; on top of this are placed large-broken stones, over that gravel, and on the top of all the natural sandy soil. The sewage is distributed over these plots by surface channels, and in such rotation that each plot works for six, and rests for eighteen hours, by which means the soil is prevented from becoming clogged, and re-aeration is established. The percolated water finds its way into the artificially-prepared channels at the bottom of the filter-bed, and thence to a water-way. The solids are incorporated with the soil. This is known as intermittent downward filtration.

Conservancy Methods.—Whether or not a water-borne system of sewerage prevails in any populous place, a considerable amount of solid refuse from ash-pits, &c., remains to be disposed of. In place of ash-pits a daily dust-bin service has been established in many towns. In smaller places, where the privy-system obtains, and where the water-supply is obtained from shallow wells often in proximity to these offices, pollution of the water sooner or later occurs with serious consequences to the consumers. In connection with farms and milk-supplies this is a matter of the greatest moment, since nothing in sanitary science has been more clearly demonstrated than that milk is probably the most common vehicle of enteric fever. In Glasgow, alone, during the past twenty-five years, almost every epidemic of this disease has been traced to in-brought milk from insanitary farms. Where the ash-pit and privy system prevails the contents should be removed at short intervals. Earth is one of the most efficient of absorbents and deodorants, hence the earth-closet is of practical value in such districts. In isolated country-houses, fitted up with modern sanitary conveniences, the usual ultimate destination of the sewage is either a cesspool or a small stream. The latter outlet is wholly objectionable. A cesspool built of brick, set in cement, and founded on concrete, at some distance from the house, in a field or in the garden, is a more preferable destination, for, in the latter case, its contents can be used for horticultural purposes, and, in the former, irrigation may be carried out.

In populous places the problem of the disposal of house and ash-pit refuse, street sweepings, offal, and miscellaneous débris, has been solved by the establishment of depôts, wherein manurial products are collected and sold, and ash-pit and other refuse burned in destructors. In this way hundreds of thousands of tons are got rid of yearly. Such works, too, may be carried on at comparatively small expenditure. All the power for steam and electric-lighting in the works is generated from the cinders from the ash-pits; the solder recovered from tins and the manure are sold, in this way reducing expenditure. With smoke-consuming apparatus, such a work can be carried on with no offence to the neighbourhood. In smaller towns, such refuse is usually carted to some piece of common land in the vicinity, and, in the open, is there set on fire, but the smoke which results therefrom is a distinct nuisance, being very malodorous.

Hints to Householders and Nurses Respecting the Disposal of House-refuse.—In the first place, nothing except ashes, soot, and inorganic débris should be consigned to the ash-pit. From the mistaken practice of throwing food-waste into that receptacle, a plague of rats is apt to follow. This may be avoided by following the foregoing advice. In the second place, food-scraps, which cannot be charitably disposed of, ought to be consumed in the kitchen fire; and in the third place, by sifting cinders, and by drying food-débris, economy may be effected in the cost of heat-production, and the bulk of the ash-pit refuse considerably lessened.

In respect of the use of disinfectants in closets for the disinfection of infective discharges from patients, it should be remembered that strong solutions of corrosive sublimate ought never to be put into that receptacle, or into slop-closets, or sinks, unless and until they are abundantly diluted after the disinfectant has had time to do its work. This disinfectant, as also chlorinated lime, attacks the lead-pipes and solder. Copious flushing should always follow. Where a privy or earth-closet takes the place of a water-closet, the disinfected discharges of a patient suffering from either enteric fever, cholera, or diphtheria, should never be placed in either, but should be interred in the soil in a remote part of a garden, or other available piece of ground.

### Midwifery Papers.

### VII.—PRESENTATIONS.

The term "presentation" is used in midwifery to denote the manner in which the fœtus enters the pelvis during labour, and is born. The position in which the fœtus enters gives its names to the presentation. Head presentations are most frequent. There are four positions in which the head can enter the pelvis: (1) Left occipito-anterior, the occiput pointing to the mother's left hip-joint, and the forehead to the right sacro-iliac joint; (2) right occipito-anterior, the occiput pointing to the right hip-joint and the forehead to the left sacro-iliac joint; (3) right occipito-posterior; and (4) left occipito-posterior, the occiput pointing to the left or right sacro-iliac joint and the forehead to the right or left hip-joint in front. In the first and second position after flexion and descent occurs, the occiput rotates forward one-eighth of a circle into the antero-posterior diameter before the head is born; in the third and fourth position the occiput has to rotate forward three-eighths of a circle into the same diameter. The head generally enters the pelvis in the oblique diameter, and the position takes its name from the part towards which the occiput points. In the first and third position the long diameter of the head (the occipito-frontal) lies in the right oblique diameter of the pelvis, but the position of the occiput is reversed in each; in the first the occiput is in the front, and in the third it lies behind in the pelvis. In the second and fourth positions the head enters in the left oblique, the occiput lying in front in the second and at the back in the fourth. In either of these positions the sagittal suture will be found running obliquely across the pelvis, and the position of the fontanelles will assist the diagnosis. In the first and second positions the large anterior fontanelle will be found lying backwards and the triangular posterior fontanelle forwards; in the third and fourth the anterior fontanelle lies forward and the posterior backwards. The caput-succedaeum is an œdematous swelling formed by the contraction of the uterus squeezing the blood and lymph into the presenting part of the head. In tedious labour, when the head is long subjected to pressure, the caput becomes large and brawny, and sometimes is an obstacle to diagnosis. Face to pubes presentations occur when the head has entered the pelvis in the third or fourth position, and the rotation forwards of the occiput three-eighths of a circle has not taken place; the forehead rotates forward one-eighth of a circle into the antero-posterior diameter, and the face is brought under the pubic arch instead of the occiput. The labour is generally delayed in these cases, which are rare.

Breech births occur next to head presentations in frequency. The buttocks of the child descend instead of the head, and are born first. In vaginal examination the head is missed, and the buttocks may be felt. After the membranes are ruptured, the finger may be inserted into the anus or the genitals may be felt. Abdominally the head may be felt at the fundus. It is very important to ensure full dilatation of the os in these cases, so keep the membranes intact as long as possible. Examine very gently. Owing to its softness the breech does not dilate the cervix so quickly as the head, and the labour is generally retarded. The risk of delay is not great to the mother, but the dangers to the child are increased from (1) the pressure of the hard head on the cord at the pelvic brim; (2) jamming of the placenta between child's head and uterine wall; or (3) the separation of the placenta from the maternal site, and the delay of the birth of the head, may cause asphyxia from stoppage of communication between the mother and child. Be prepared to do artificial respiration, having a hot bath and assistance in readiness. After full dilatation and rupture of membranes have taken place, give full time for the breech to be born, and never pull on it. When the body is born as far as the navel, release the feet and pull down a loop of the cord to prevent it dragging, placing it in the oblique diameter of the pelvis, where it will get least pressure from the after-coming head. When the shoulders are born, direct the child's body forwards between the mother's thighs, being careful not to use traction. The midwife's left hand should firmly grasp the fundus during the progress of labour. The head is invariably delayed by failure of flexion in breech cases, so be prepared to assist its birth by passing two fingers of the right hand up the child's back and press on the occiput, or two fingers of the left hand (the right grasping the fundus) may be passed up the front of the child and inserted into the mouth, and flexion of the head induced, but the former is far the best plan. Sometimes, instead of the child's arms being placed over the chest, they are extended over its head. Release the arms one by one by passing two finger up the child's back to the forearm and pushing the arm over the face. Make pressure on the fundus to assist the birth of the head, and to prevent the extension of the arms. In a breech presentation, if there are strong pains, and the presenting part makes no advance, send for a medical man, as the breech may be jammed.

Footling and knee presentations occur very rarely, and their treatment is very much the same as in breech cases. Face-presentations are caused by the head entering the pelvis *extended* instead of flexed on the chest. The chin descends first instead of the occiput, and rotates forward into the antero-posterior diameter, and then advances under the pubic arch, when flexion brings the forehead and rest of the head over the perineum. In face-presentations the face is generally bruised by the uterine pressure during labour, and the caput-succedaeum is found on the upper part of either cheek, enclosing the outer edge of the orbit. The diagnosis of face-presentation is difficult, and is sometimes mistaken for breech. After the rupture of the membranes, the prominence of the nose with the orifices of the nostrils, and the mouth with its hard ridges of gums can be felt. The edges of the orbits can be felt, but great care should be taken not to injure the eyes in examining. In the management of the labour keep the membranes intact as long as possible during the first stage, but in the second stage, if the chin has not rotated, but is found low down in the hollow of the sacrum, send for a medical man, as his help will be required. In this presentation the danger to the mother is exhaustion from protracted labour. The danger to the child is great if the head gets jammed and rotation does not take place. The pressure upon the child's face and vessels of the neck often cause bruising and swelling, and sometimes asphyxiation.

Brow presentation.—In this presentation the head enters the pelvis neither flexed or extended. By vaginal examination the rounded surface of the frontal bone can be felt, and the anterior fontanelle can be reached in one direction, and the orbit and the root of the nose can be felt in another. Send for a medical man, as assistance will be needed.

Transverse presentations.—The long diameter of the child lies across the uterus. The presenting part may be the shoulder, arm, or elbow; the head is found in one or other of the iliac fossæ. Keep the membranes intact as long as possible, and send at once for a medical man. A contracted pelvis is the principal cause of transverse presentations.

Funis presentation or prolapse of the cord often occurs in transverse presentation. The danger to the child is great of death from asphyxia when the cord is prolapsed. Prolapse is caused by any condition which prevents the complete filling of the lower part of the uterus. Prolapse occurs in placenta prævia, excess of liquor amnii, long cord, or cord attached to lower margin of placenta. When the cord is prolapsed before the membranes are ruptured, place the patient in the knee and elbow posture, and the cord sinks down behind the child's head by action of its own weight. If the membranes are ruptured, replace the cord in the uterus above the presenting part, securing it as far as possible from pressure. In funis presentations it is always better to send for a medical man's help, as the danger to the child is great.

### Nurses in 1896—Their Quarters, Hours, and Food.

### GREAT NORTHERN CENTRAL HOSPITAL.

### I.—Terms of Training.

The training offered to nurses at this hospital is especially valuable, combining as it does the nursing of private patients, for whom special arrangements are made, with ordinary experience in the general wards. Candidates must be between the ages of twenty-three and thirty-five, of average height and physique. Those selected are taken on trial for two months, at the end of which time they are, if suitable, engaged as probationer nurses, signing for a period of three years, dating from the day of entrance into the hospital. Certificates are given at the completion of three years' satisfactory service.

Regular courses of lectures are given by members of the medical staff on medical, surgical, and obstetric nursing. At the end of each course probationers are required to pass an examination, failure in which may cause dismissal for inefficiency, or postpone the granting of the certificate. Attendance at lectures does not count as off duty time. Owing to the terms of training at the Great Northern Central Hospital, which demand from probationers a year's gratuitous work, besides a fee of £10, the nurses for the most part belong to a superior social class. They are generally the daughters of professional men, tradespeople, or farmers, and are well educated.

### II.—Hours of Work and Times Off Duty.

Nurses and probationers go on duty in the wards at seven a.m., leaving them at night at nine o'clock. During these fourteen hours two hours are daily allowed off duty, with one half-hour after the morning's work for dressing and luncheon and half an hour each for dinner and tea, leaving on actual duty ten and a-half hours. Night nurses and probationers are on duty from nine p.m. to half-past eight a.m., with time for meals, and—an excellent practice rarely in use—they are allowed in addition one half-hour off duty, during which time the night superintendent relieves them. A supernumerary probationer is on duty at night, attached to no special ward, but ready to go where work is heaviest. Night nurses go out for two to three hours every day, in the morning in winter in the evening in summer. A day off, from seven a.m. to ten p.m. is allowed once a month, and a fortnight's leave in the year. At the end of the probationers' second year they are allowed three weeks' holiday. Sisters are on duty from seven a.m. to nine p.m., having half an hour for lunch in the morning, three-quarters of an hour for dinner, and half an hour for tea. In most hospitals it is usual for the ward sisters to begin duty an hour later than the nurses and probationers. Here, however, they have the same number of hours on duty as the rest of the staff, except that they have a quarter of an hour longer allowed for their dinner. The times off duty are nominally two evenings in the week from six to nine, and two afternoons from four to six, with every other Saturday from two to ten ; a day once a month "when practicable," and four weeks' holiday in the year. Practically it may be understood that leave to go out is given when desired, and the sisters' hours off duty must necessarily be a little more elastic in arrangement than those of the nurses and probationers. At the Great Northern Central Hospital the absence of a ward sitting-room renders it impossible for the sister to take advantage of momentary pauses in the work of the day, and so she is actually on duty in the ward the whole time she remains in it.

Miss Hull takes much interest in the well-being of her nurses, and arranges carefully for their comfort. Special permission has to be obtained to sleep away from the hospital, but leave is given when convenient. The whole benefit of days off is secured to the nurses as they do not come on duty at all on that morning.

### III.—Meals.

Day nurses' breakfast at half-past six a.m., at which meal they have eggs, bacon, or fish, tea and coffee. A light luncheon, milk and bread and butter, they have when they come off duty for dressing in the morning, about half-past nine. Dinner is at half-past twelve and one p.m., for which hot roast meat, rabbit pies, vegetables, and pudding are provided. Tea is laid in the dining-room from four to five p.m. ; supper is at nine p.m., for which hot or cold meat and pudding are provided. No alcohol is allowed the nurses, except by doctors' orders. Milk and lemonade are always on the table. Night nurses have supper (or breakfast, as it is called in some hospitals) at half-past eight p.m., for which they have soup, eggs, or meat stew ; they take with them to the wards eggs or cold meat, with bread and butter, for the night meals, this food being kept in a specially-contrived little larder in the corridor outside the ward, which is well ventilated from the outer air.

### IV.—Salaries and Uniform.

Probationers at the Great Northern Central Hospital are not paid during their first year, and they are required to pay a fee of £10 if they are accepted after the first two months' trial. After the first year they receive a salary of £15 for the second year and £20 for the third year. Sisters are paid a salary of £28 a year, rising by £1 annually to £35. Uniform, consisting of three print dresses and caps, and nine aprons, is supplied by the hospital. An allowance is made for washing, each nurse being entitled to send to the laundry twenty pieces a week. Outdoor uniform is not obligatory.

### V.—Nurses' Quarters.

The nurses' rooms at the Great Northern Central Hospital are in the main building. They are for the most part single rooms, and are comfortably furnished. There are four rooms which contain two beds each, divided by screens. Sisters have bedrooms away from the wards, and these have each an easy chair and a fire-place, so that they may be used for sitting in, if their occupants prefer this to making use of the general sitting-room. This is a comfortable room divided from the general dining-room by folding doors, but both are really too small, and more accommodation is needed for the staff in this respect. With regard to sisters' rooms off the wards, which here do not exist, a diversity of opinion prevails among hospital authorities ; the general feeling amongst matrons and ward sisters, however, is in favour of sitting-rooms adjoining the ward, and it is certain that the responsible head of a large ward does need some quiet corner near her work, where she may retire for an occasional few minutes when her presence is not urgently needed in the ward. There are many half-hours, for instance, of waiting for the doctors, which might profitably be spent by a hard-working sister in resting in her own room. Where this is in another part of the building, such retirement is naturally impossible. Nurses who may chance to be ill at this hospital are comfortably cared for in the private wards, an arrangement which renders a special sick-room unnecessary.

### Where to Go.

The Secretary requests us to state that a course of demonstrations on "Invalid Cookery" will be given during the winter months at the offices of the Association, commencing in November. The terms will be as follows: For the full course of ten demonstrations, members 7s. 6d., non-members 12s. 6d. (these tickets will be transferable) ; single demonstrations—members 1s., non-members 1s. 6d. Syllabus and further details can be obtained from the offices of the Association, 17, Old Cavendish Street, London, W. H.R.H. Princess Christian has graciously expressed her intention of attending some of the demonstrations.

### The British Nurses' Association.

BREAY v. BROWNE.

In the City of London Court, on Monday, the case of Breay v. Browne was decided before Mr. Commissioner Kerr and a jury. The case raised questions of much interest to the medical and nursing professions. The proceedings were brought by Miss Margaret Breay, Inglewood, Fleet, Hants, and she sought to recover the sum of £50 as damages against the defendant, Sir James Crichton Browne, Carlisle Mansions, Victoria Street. Mr. Scarlett (instructed by Messrs. Mear and Fowler) was counsel for the plaintiff, and Mr. Muir Mackenzie for the defendant. Mr. Scarlett said the action was of immense importance to the nursing profession, as it affected the rights of nurses to govern their own association. The plaintiff was a member of the Royal British Nurses' Association, and she had been so since 1887, when she was matron of the English Hospital at Zanzibar. The defendant was one of the vice-presidents of the association and a member of the governing body. What the plaintiff complained of was that she had not been allowed to exercise her rights as a member of the association owing to illegal, improper, and wrongful refusal on the defendant's part to submit a resolution to the meeting of the association which was held on July 22, at St. Bartholomew's Hospital. The defendant with others was endeavouring to get the nurses out of the association, and the medical men wanted to have the control of the association. That was not intended when the Royal Charter was granted to the association, as it provided that there should be 100 matrons, 100 nurses, and 100 medical men to compose the executive council. The medical profession, with the defendant at the head, were endeavouring to swamp the nurses from the Association, and the plaintiff had brought that action not only on her own behalf but also for the sake of the other members, as she wished to have it declared that there was no reason why she should not exercise her rights as a member and have her resolution submitted to the meeting. The only reason why the defendant did not submit the resolution to the meeting was because it was not sent by registered post. The plaintiff was called, and she bore out her learned counsel's statement. Mr. Commissioner Kerr said he could not understand why the defendant did not allow the resolution to be submitted to the meeting. Sir James Crichton Browne, in his evidence, said that he had no ulterior motive, as had been suggested, in not submitting the plaintiff's resolution to the meeting. Until he arrived at the hospital he had never even heard of the plaintiff's existence; and he did not even know that she was going to submit a resolution. When the resolution was brought up an objection was made on the ground that all the formalities had not been observed as provided by the bye-laws. The resolution had not been sent by registered post; and he was of opinion that that was fatal to the matter being proceeded with. As a matter of fact, the resolution was a censure upon himself; and if he could have had the whole question gone into and an explanation given, thus clearing the grievance up, he would have been only too glad to have assisted in such a desirable matter. He simply had the interests of the association at heart. The meeting was of a very stormy character, and there were threats of injunctions and mandamuses against the association. All he wanted was to prevent litigation, and that was why he would not allow anything to be done which was not strictly in accordance with the bye-laws. There was no ground whatever for the suggestion that he was guilty of any partiality or wrongful act towards the plaintiff. It was true that she sat amongst the turbulent members, but that circumstance did not influence his mind in any way. Several witnesses were called to testify to the defendant having shown no partiality at the meeting. After the learned counsels' addresses, Mr. Commissioner Kerr summed up. He said he thought it was very unfortunate that the defendant did not submit the resolution to the meeting, despite the question as to how it arrived at the offices of the association. The jury had simply to say whether or not the defendant acted from indirect motive. The jury, after a few moments' deliberation, said they found for the plaintiff with ¼d. damages. Mr. Mackenzie asked for judgment for the defendant on the ground that there was no ground of action to justify the jury's verdict. Mr. Commissioner Kerr said he would consider how he would enter judgment. He would give costs on the higher scale, as the case was so very important, and he would give the unsuccessful party leave to appeal.

### Appointments.

MATRONS.

Luton Infectious Hospital.—Miss M. L. Thomas has been appointed Nurse-Matron at this hospital. She was trained at the City Infirmary, Birmingham, where she was afterwards promoted to the position of nurse in charge. She has held a similar appointment at the Monsall Fever Hospital, Manchester, and has worked at the Women's Hospital, Liverpool, and at the North-Eastern Fever Hospital, London. Miss Thomas has also acted as Matron at the Borough Fever Hospital, Bury, Lancashire.

Borough Infectious Hospital, Ashton-under-Lyne.—Miss Margaret Brewester has been appointed Matron to this hospital. Miss Brewester was trained at the Royal Southern Hospital, Liverpool, with which institution she was connected for seven years as a private nurse, occasionally taking sister's duty in the wards. For the past three years she has held the appointment of sister in charge of the children's wards at the Royal Albert Edward Infirmary, Wigan. Sister Brewester carries with her to her new work many good wishes from her fellow-workers at Wigan.

### Minor Appointments.

Miss Emma Evans has been appointed Superintendent night nurse at the Stockport Workhouse Infirmary. Miss Evans was trained at St. Mary's Hospital, Manchester, and comes to her new work from Sculcoates Union Infirmary, where she has held the post of superintendent nurse.

Western Fever Hospital.—Miss Ethel Jessie Atkins has been appointed Assistant Matron at this Metropolitan Asylums Board Hospital. She was trained at St. Bartholomew's Hospital, has acted as charge nurse at the South-Western Fever Hospital, and has lately gained further experience in fever nursing as sister-in-charge of a pavilion at the City Hospital, Birmingham.

Fir Vale Infirmary, Sheffield.—Nurse A. S. Pruett, who for the last two years has taken charge of the male surgical wards at this infirmary, has been appointed Night Superintendent of Nurses. Nurse Pruett was trained at the Royal Infirmary, Glasgow, and worked for six years for the Workhouse Infirmary Nursing Association. She holds the L.O.S. diploma.

Mill Lane Hospital, Liscard, Cheshire.—Miss C. A. Snelson, Miss Lucy Osborne, and Miss Emily Ainsworth have been appointed Charge Nurses at the above hospital. Miss Snelson was trained at the Royal Southern Hospital, Liverpool, where she worked for nearly five years. She also holds testimonials from the Fever Hospital, Douglas. Miss Osborne was trained at Chichester and Preston Hospitals, and has since worked at the Yarmouth Infectious Hospital, the Harrogate Institute, the West Malling Nurses' Institute, and the Bolton Nurses' Institute. Miss Ainsworth received her training at the Salford Royal Hospital, and has since held appointments at the City of London Hospital, Victoria Park, E., and at the Salford Union Infirmary.

### Everybody's Opinion.

### HOMES FOR THE DYING.

Miss Florence Smedley, Lady Superintendent, Hospital for Sick Children, Great Ormond Street, writes In The Hospital for September 26th you have an interesting account of "Friedenheim" and the "Hostel of God." You also make reference to a third home in Osnaburgh Street. Those of your readers interested in the matter may care to know something of the work of the Sisters of St. Peter at their home in Mortimer Road, London, N.W., and at Cheddar. Since the foundation of St. Peter's Home, in 1861, a ward has been set aside there for the dying. Those patients whom no hospital can keep are received by the Sisters and nursed with the tenderest care. St. Michael's Home, Cheddar, which was built and endowed by Mrs. William Gibbs for twenty-two men and fourteen women, is also under the care of the community of St. Peter. St. Michael's Home is reserved almost exclusively for patients in the advanced stage of phthisis. I feel sure that the Sisters will welcome any visitor to their home who would care to know more of this work.

### THE LEICESTER "NURSES' CO-OPERATION."

"A Private Nurse" writes: In the interests of the private nurses of Leicester, it seems to me well that the public should have a clearer idea of this "Nurses' Co-operation" than is to be gained by the notice in your paper of last week's issue. It is composed of a number of nurses who, after having joined an "Association of Nurses," split because they considered that they should have exactly the same advantages as the original three who have borne the expense and risk of starting an association on co-operative principles. The nurses joining the "Association," I believe, were required to pay either £8 or £8 10s. a year as their share towards rent of house, coals, gas, &c., besides having the cases found for them. To this some could not agree, so have hired another house, calling it the "Nurses' Co-operation." Training is somewhat scanty amongst them. Some may have had two years' training, others for certain one year, ten months, and one three months in a general hospital. The committee would appear to consist of these, which, of course, speaks well for their power of nursing organisation. The "Matron" was housekeeper to the original "Association," and untrained. Neither of these have any connection with the old-established "Institution of Trained Nurses," Aylestone Road, Leicester.

The Superintendent of the "Association of Trained Nurses," 45, Princess Street, Leicester, writes: In your issue of The Hospital dated September 26th, reference is made to the Nurses' Co-operation in this town. We take the liberty of pointing out the circumstances out of which the newly-formed organisation has arisen, together with its object. The idea itself is by no means original in Leicester, where the "Association of Trained Nurses" has been in active operation for nearly four years, finding constant employment for fifteen duly qualified nurses, all of whom received full remuneration for their work, with the exception of a small annual amount from each nurse towards defraying the expenses of a comfortable and pleasant home. The history of the "Association" may, perhaps, interest your readers. In the year 1892 five members of the Leicester Nursing Institution, who had been on the staff six to twelve years, considered their position unsatisfactory, and wishing to have the nursing fees themselves started the present Nurses' Association. Within the last few months some little unpleasantness arose regarding its management, resulting in the matron and eight of its members leaving, who now form the Nurses' Co-operation. The rules of the "Association" are few, similar to those of other institutions of the kind; four of the older nurses take all responsibility and form the executive. The Association of Nurses have secured a lady superintendent in the person of Miss Slade, who for eight years held a similar position in the Leicester Institution of Trained Nurses, Aylestone Road.

*** It would appear from the above letters that the so-called Nurses' Corporation at Leicester is not managed by an independent committee, an important fact for nurses to note and remember.—Ed. *T. H.*

### Nursing in America.

We alluded in a recent number to the scheme described in the following account received from Miss Dock, which we now give in full:—

"On September 2nd a convention of nurses met at Manhattan Beach Hotel to consider the formation of an articulated association of graduate nurses, which will ramify throughout the United States and Canada. This assemblage of representative nurses was one of a succession of movements extending back over several years' time, and all directed towards this end. The first step taken was in June, 1893, when, at the time of the World's Fair in Chicago, representatives of training schools met in the Hall of Columbus to organise an Association of Training School Superintendents.

"The chairman of the Nursing Section of the Medical Congress, Miss Hampton (now Mrs. Robb) foresaw the rise of an association of nurses, and at that time pointed out the important part that organised Alumnæ societies would play in its development, and advised that they be fostered and multiplied.

"At the third annual meeting of the Association of Superintendents of Training Schools in Philadelphia in February, 1896, a paper on "A National Organisation" was read, and a committee appointed to see if the time was ripe for its initiation. The committee numbered five, namely: Miss Merritt, superintendent of the Brooklyn City Training School; Miss Walker, of the Pennsylvania; Miss McIsaac, of the Illinois; Miss Brown, of the Massachusetts General; and Miss Dock, who was the secretary of the American Society of Superintendents of Training Schools, was appointed chairman. To these were added Miss Draper, of the Royal Victoria; Miss Snively, of the Toronto General; Miss Maxwell, of the Presbyterian, New York; Miss Hutcheson, of St. Luke's, Chicago; Mrs. Robb; Miss Palmer, of the Rochester City; and Miss Nutting of the John Hopkins. This committee, representing the Superintendents' Society, following the instructions given them by that society, chose twelve of the most generally representative Alumnæ Associations, and a letter was written to the secretary of each one asking that the subject of the formation of a national association be considered, and that a delegate be chosen from among the Alumnæ members not holding hospital positions to meet in convention the representatives of the Superintendents' Society, and draft a constitution upon which to organise. All the Alumnæ Associations written to responded with interest, and in the affirmative. Elections were held promptly, delegates appointed, and their names sent in. A short correspondence resulted in fixing time and place. The time was set early, in order that the results of the work of the convention may be laid before the various Alumnæ for ratification, their actions taken, and a report prepared for the next annual meeting of Superintendents in February, 1897.

"The delegates appointed to represent the Alumnæ Associations were as follows: Farrand Training School Alumnæ, Miss Mary E. Smith; Illinois Training School Alumnæ, Miss Phœbe W. Brown; Massachusetts General Alumnæ, Miss M. W. Stevenson; New Haven Training School Alumnæ, Miss Ella Clapp; Bellevue Training School Alumnæ, Miss Rhodes (substitute, Miss Warren); New York Training School Alumnæ, Miss Lena A. Walden; Brooklyn City Training School Alumnæ, Miss Laura Healy; Orange Memorial Training School Alumnæ, Miss Margaret Anderson (substitute, Miss Bessie Pierson); Philadelphia Training School Alumnæ, Mrs. J. R. Hawley; University of Pennsylvania Training School Alumnæ, Miss H. Morand; Johns Hopkins Training School Alumnæ, Miss Helena Barnard (substitute, Miss Ross); Garfield Training School Alumnæ, Miss M. A. Mullen. The roll-call on the first day showed fifteen members present, of whom ten were Alumnæ delegates, and at later sessions the two others were present at least once. The sessions lasted for three days; at the end of this time, a constitution and bye-laws having been satisfactorily drafted, the convention adjourned, to meet in February, 1897."

### For Reading to the Sick.

THE LOVE OF GOD.

**Motto.**

"God is Love."—1 *John* iv. 8.

**Verses.**

God only knows the love of God;
O that it now were shed abroad
In this poor stony heart!
For love I sigh, for love I pine;
This only portion, Lord, be mine,
Be mine the better part.

Yet I may love Thee, too, O Lord,
Almighty as Thou art,
For Thou hast stooped to ask of me
The love of my poor heart.
—*Hymns Ancient and Modern.*

There are who sigh that no fond heart is theirs,
None loves them best. O vain and selfish sigh!
Out of the bosom of His love He spares—
The Father spares the Son, for thee to die;
For thee He died—for thee He lives again;
O'er thee He watches in His boundless reign.

Thou art as much His care, as if beside,
Nor man nor angel lived in heaven or earth;
These sunbeams pour alike their glorious tide
To light up worlds, or wake an insect's mirth;
They shine and shine with unexhausted store—
Thou art thy Saviour's darling—seek no more.—*Keble.*

My cup hath long run o'er
With blessings crown'd—many and multiplied;
And daily, from the fount of Love supplied,
Upon my head they pour.

**Reading.**

Strange it is that love should be one attribute that man shares with the Highest of all as well as with the lower creation. Faith and hope belong to a man as being a partly spiritual and partly material creature: in one sense finite, in another sense infinite; but in love alone can he resemble God; by love alone can he know anything of the nature of God; and at the same time it is only the very lowest animals of all who are (so far as we know) incapable of love of offspring, while the superior ones among them often show a most touching and pathetic force of affection to one another and their masters.

And we may observe that just as the animals that rise to the noblest class of instinct carry their affections beyond the love of offspring and of mate, so the more degraded mankind become, the more these better natural feelings become lost, till even maternal love is extinguished in selfishness beneath that of the brutes. . . . Loves comes from God, and returns to Him. Therefore it has in it no weakness of mere tenderness, and understands the royal law of doing to others as we would be done by.

One who loves God cannot bear to see Him offended, and thus is zealous to reprove, correct, and, above all, to pray for the offender. It is not possible to enter fully into the glories and graces of that love to God and love to man in Him which is, in fact, the proof and action of faith and hope, the means of growing in both here below, and the survivor of both, living and expanding for ever in the eternity where they will be no longer needed.—*Charlotte M. Yonge.*

I love them that love Me, and those that seek Me early shall find Me.—*Prov.* viii. 17.

We love Him, because He first loved us.—1 *John* iv. 19.

### Notes and Queries.

*Nursing Abroad.*

(11) I am very anxious to get a post as nurse abroad; can you help me? I am hospital trained, and have had a good deal of experience also in private work.—*Nurse F. D.*

Do you look in our advertisement columns each week, or have you put an advertisement in yourself, stating the sort of post you require? To do this will be your best chance. You might write to Mrs. Dan Norris, Villa d'Ormesson, Cannes, who has a very successful private nursing home, and might be able to help you.

*Massage.*

(12) Please tell me of what the Weir Mitchell treatment consists, and how to massage the spine?—*F. S.*

You had better read Mrs. Creighton Hale's "Art of Massage" (Scientific Press, 28 & 29, Southampton Street, Strand, London, W.C.), but you cannot learn massage from books. If you want instruction write, enclosing stamped envelope, to the Hon. Secretary, Society of Trained Masseuses, Trained Nurses' Club, 12, Buckingham Street, Strand, asking if she will kindly give you advice.

*One Year's Training.*

(13) Would you give me the names of some hospitals in London or the country where non-paying probationers would be received for one year's general training?—*Constance.*

We do not think you will find a general hospital of any standing either in London or the country that will take non-paying probationers for so short a term. Some few institutions still give two years' training, but in most cases a three years' term of service is required. You will find the names of hospitals, and the terms upon which training is given, in "How to Become a Nurse" (Scientific Press, 28 & 29, Southampton Street, Strand, London, W.C.).

*St. Bartholomew's Hospital.*

(14) Please tell me if an article on St. Bartholomew's Hospital has yet appeared in the series called "Nurses in 1896," and if so, can I get the number containing it from The Hospital Office? Also, can you recommend me a book on physiology which would be sufficient preparation for the St. Bartholomew's entrance examination?—*G. B.*

Yes; the article on St. Bartholomew's Hospital appeared in The Hospital for September 12th. Write for the number to the Manager, The Hospital Office, 28 & 29, Southampton Street, Strand, London, W.C., enclosing 2½d. in stamps. Dr. Percy Lewis's "Nursing: Its Theory and Practice," published by the Scientific Press, 28 & 29, Southampton Street, Strand, London, W.C., will be the most useful book for your purpose.

*Formaldehyde.*

(15) Can you tell me where I can get full particulars of the antiseptic Formaldehyde, on which several papers have appeared in The Hospital; where it can be obtained, and where it has been used?—*E. M. V.*

The best account of the methods and results of disinfection by Formaldehyde are to be found in the annals of the Pasteur Institute of this year, we believe in the numbers for April and May. References will also be found in the *Centralblatt fur Bacteriologie* for the same period of the uear. Formalin, which is a 40 per cent. solution of Formaldehyde, and is the form in which it is generally used, can be obtained from any wholesale chemist. The autoclave, or apparatus by which the spray is produced, can be obtained from Messrs. Baird and Tatloch, London; and Messrs. Hawksley and Son, London. We do not think the method has yet been tried in England in the disinfection of houses, but it has been used abroad both in Paris, Montpelier, and Berlin.

*Monthly Nursing.*

(16) I am going in for monthly nursing very shortly, and in the meantime should be glad if you would tell me the best books to study.—*Amy L.*

"A Short Manual for Monthly Nurses," by Dr. Cullingworth, published by J. and A. Churchill, price 1s. 6d. Write to the matron of the school where you intend to enter as a pupil, and ask her advice.

*Training.*

(17) Please tell me (1) if it is possible to be trained as a nurse without paying a premium; and (2) what steps should one take to gain such training?—*M. J.*

(1) Certainly. (2) Read "How to Become a Nurse" (Scientific Press, 28 & 29, Southampton Street, Strand, London, W.C.), where you will find full details about the terms of training at London and provincial hospitals. Then write to the matrons of the various institutions for forms of application.

*Nursing in Alexandria.*

(18) How can I get work as a nurse in Alexandria?—*Nurse E.*

Do you mean as a private nurse or in the British hospital? You might apply to the matron of the latter, and ask her advice regarding private nursing prospects, but on no account go abroad without a certain engagement.

*Nursing in Shanghai.*

(19) I should like to go to Shanghai as a nurse. Is there likely to be a vacancy.—*Margaret M.*

The nurses who have gone to Shanghai were selected by Mr. H. C. Burdett at the invitation of the municipality of Shanghai, and no further staff is likely to be required for some time. We take this opportunity to point out that when an application is made full qualifications should be stated, and copies of testimonials enclosed. Applicants are very apt to forget these essential details, to their own detriment.

*Training.*

(20) Please give me the addresses of some hospitals, children's or otherwise, where I could be admitted as probationer at the age of 18. What is the price of "How to Become a Nurse"?

No general hospitals admit such young probationers. You will find in "How to Become a Nurse" (Scientific Press, 28 & 29, Southampton Street, Strand, London, W.C.; price 2s. 6d.), the addresses of children's hospitals, and the ages at which candidates are eligible—usually from 18 or 20.

